# Aminoacyl-tRNA Synthetase: A Non-Negligible Molecule in RNA Viral Infection

**DOI:** 10.3390/v14030613

**Published:** 2022-03-15

**Authors:** Min Feng, Han Zhang

**Affiliations:** Institute of Medical Biology, Chinese Academy of Medical Sciences and Peking Union Medical College, Kunming 650118, China; fengmin@imbcams.com.cn

**Keywords:** aminoacyl-tRNA synthetase, multi-synthetase complex, aaRS-interacting multi-functional protein, coronavirus, RNA virus, innate immune response

## Abstract

Infectious diseases such as the ongoing coronavirus disease 2019 (COVID-19) continue to have a huge impact on global health, and the host-virus interaction remains incompletely understood. To address the global threat, in-depth investigations in pathogenesis are essential for interventions in infectious diseases and vaccine development. Interestingly, aminoacyl-transfer RNA (tRNA) synthetases (aaRSs), an ancient enzyme family that was once considered to play housekeeping roles in protein synthesis, are involved in multiple viral infectious diseases. Many aaRSs in eukaryotes present as the components of a cytoplasmic depot system named the multi-synthetase complex (MSC). Upon viral infections, several components of the MSC are released and exert nonenzymatic activities. Host aaRSs can also be utilized to facilitate viral entry and replication. In addition to their intracellular roles, some aaRSs and aaRS-interacting multi-functional proteins (AIMPs) are secreted as active cytokines or function as “molecule communicators” on the cell surface. The interactions between aaRSs and viruses ultimately affect host innate immune responses or facilitate virus invasion. In this review, we summarized the latest advances of the interactions between aaRSs and RNA viruses, with a particular emphasis on the therapeutic potentials of aaRSs in viral infectious diseases.

## 1. Introduction

Aminoacyl-transfer RNA (tRNA) synthetases (aaRSs) are an ancient enzyme family that links specific amino acids (AA) onto their cognate tRNAs in a two-step reaction. The first step is to form an aminoacyl-adenylate intermediate (AA-AMP) while releasing a pyrophosphate (PPi). The second step is to yield the aminoacyl-tRNA by transferring AA-AMP to the cognate tRNA while releasing AMP [1].

In humans, two forms of aaRSs exist in cytoplasmic (cy) and mitochondrial (mt) protein synthesis, respectively. The cy-aaRSs in higher eukaryotes are further divided into two groups: some remain free in cytoplasm and the others are the components of the multi-synthetase complex (MSC). Although the function of aaRSs in translation and protein synthesis is well recognized, increasing evidence has proved their roles in viral infection. For example, some aaRSs are released from the MSC to exert nonenzymatic activities upon infection, whereas some aaRSs are utilized by viruses for internalization, package, and replication. In addition, some aaRSs are even secreted as active cytokines to modulate host innate immune responses. These discoveries pinpoint that aaRSs act as an essential cellular sensor and immunoregulator upon infection. These ex-translational roles are mainly attributed to the evolutionary changes of the mammalian aaRSs by appending new domains or motifs (except for alanyl-tRNA synthetase), such as the glutathione S-transferase domains, the WHEP domains, the leucine zipper domains, etc. [2]. Equipped with these new domains or motifs, aaRSs behave as “molecular transformers” by reshaping their architectures or functions, thereby allowing novel roles in higher organisms [2,3].

Given the essential effects of aaRSs on viral infection, we aim to discuss the latest studies of aaRSs in viral infectious diseases, with a particular focus on the potentials of aaRSs for precise intervention or therapies in viral infection. Here, the virus exclusively refers to the RNA virus, with the other types of viruses omitted in our review unless specified otherwise. 

## 2. Potential Roles of aaRSs in Infections with Coronaviruses

Coronavirus (CoV) is an enveloped, single-stranded positive-sense RNA virus, a pathogen responsible primarily for enteric and respiratory infections in vertebrates and humans [4]. Compared with other RNA viruses, CoVs contain the largest known RNA genome whose replication and transcription are quite complicated. As such, the pathogenesis of CoVs infections and the host-CoV interaction remain unclear.

Among pathogenic human CoVs, coronavirus disease 2019 (COVID-19) is a serious multisystem disease caused by a novel CoV named severe acute respiratory syndrome coronavirus 2 (SARS-CoV-2). In addition to COVID-19, infections with SARS-CoV-1 and Middle East respiratory syndrome CoV (MERS-CoV) took place in the past, leading to acute pneumonia and higher mortality in human. To address the global crisis, a mounting number of CoVs studies on either the viral genome or the hosts have been conducted. These discoveries have revealed various molecular and cellular changes in both clinical samples and infected cells.

Very recently, we provided an overarching landscape on the correlation between aaRSs and CoVs based on available omics data [5]. Interestingly, most mt-aaRSs seem to play antiviral roles based on their Z scores across infections with SARS-CoV-1, SARS-CoV-2 and MERS-CoV, and the interaction networks between CoVs and human aaRSs also reveal a strong involvement of mt-aaRSs. Further validation experiments confirmed the physical interaction between SARS-CoV-2 main protease protein (M protein) and mitochondrial threonyl-tRNA synthetase (mt-ThrRS). Moreover, mitochondrial translation and ribosome proteins were also dramatically changed upon CoVs infections, and two mitochondrial ribosome proteins, MRPL28 and MRPL37, were found to have the same expression trend as the changes of mt-aaRSs across different datasets, suggesting a potential association between mt-aaRSs and mitochondria upon SARS-CoV-2 infection [5]. 

In fact, viruses have evolved multiple strategies to evade the host immune defense. One mechanism is that viral proteins interfere with the function of the mitochondrial antiviral signaling protein (MAVS), an adaptor that coordinates the activation of interferon (IFN)-induced pathways and autophagy at the mitochondrial level [6]. Indeed, one recent work has identified the SARS-CoV-2 membrane glycoprotein M as a factor in the inhibition of host antiviral innate immunity by directly targeting MAVS [7]. In this regard, mt-aaRSs are very likely to be hijacked by SARS-CoV-2 during infection, and their interaction leads to an inadequate innate immune response in the host by modulating mitochondria-mediated signaling. Although no in-depth mechanisms have been characterized regarding the interaction between SARS-CoV-2 and mt-aaRSs, it is worth thoroughly investigating the function of mt-aaRSs during SARS-CoV-2 infection, especially the effects of their interaction on the host immune responses.

## 3. Host aaRSs Are Involved in Host Innate Immune Responses to RNA Viral Infections

**Figure 1:**
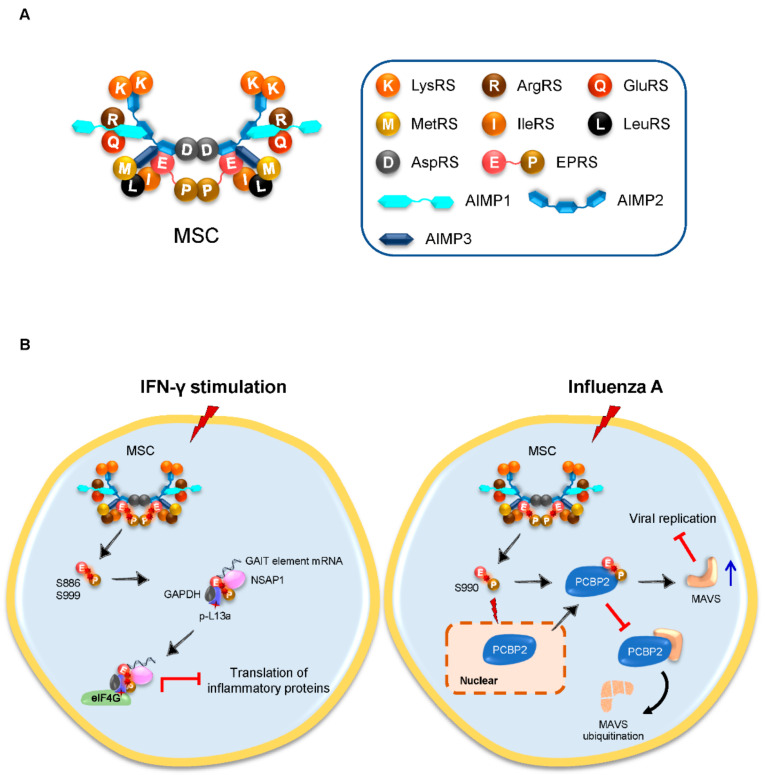
MSC and EPRS in viral infection. (**A**) Schematic representation of the MSC, which contains 9 cy-aaRSs and 3 AIMPs. The cartoon model of the MSC was generated based on previous studies [11,12,13]. AIMP, aaRS-interacting multi-functional protein; ArgRS, arginyl-tRNA synthetase; AspRS, aspartyl-tRNA synthetase; EPRS (E-P), glutamyl-prolyl-tRNA synthetase; GluRS, glutamyl-tRNA synthetase; IleRS, isoleucyl-tRNA synthetase; LeuRS, leucyl-tRNA synthetase; LysRS, lysyl-tRNA synthetase; MetRS, methionyl-tRNA synthetase; and MSC, multi-synthetase complex. (**B**) IFN-γ stimulation induces sequential phosphorylation of EPRS at serine 886 (S886) and S999 (red stars), which results in the dissociation of EPRS from the MSC. The released EPRS forms a GAIT complex with NSAP1, p-L13a, and GAPDH. The GAIT complex further represses translation of inflammatory proteins via its interaction with eIF4G (left). By contrast, influenza A induces EPRS phosphorylation at S990 (red star) and causes its release from the MSC. The released EPRS interacts with PCBP2 and blocks PCBP2-mediated ubiquitination of MAVS. The increase in stabilized MAVS (blue arrow) further suppresses viral replication (right). eIF, eukaryotic initiation factor; GAIT, IFN-γ-activated inhibitor of translation; GAPDH, glyceraldehyde-3-phosphate dehydrogenase; IFN, interferon; MAVS, mitochondrial antiviral signaling protein; NSAP1, NS1-associated protein 1; PCBP2, poly(rC)-binding protein 2; and p-L13a, phosphorylated ribosomal protein L13a.

The mammalian MSC contains 9 cy-aaRSs (glutamyl-, prolyl-, isoleucyl-, leucyl-, methionyl-, glutaminyl-, lysyl-, arginyl- and aspartyl-tRNA synthetase) [8] and 3 nonenzymatic proteins termed aaRS-interacting multi-functional proteins (AIMPs), including AIMP1, AIMP2, and AIMP3 [9,10]. Among them, glutamyl- and prolyl-tRNA synthetases (GluRS and ProRS) are coupled together as glutamyl-prolyl-tRNA synthetase (EPRS). MSC is ubiquitously expressed in cells, and its components are tightly bound to each other via noncatalytic domains (Figure 1A). The functional significance of the MSC remains unclear, but the formation of the MSC appears to facilitate specific AA charging to the tRNAs and coordinate protein synthesis [8]. Beyond its catalytic roles, MSC is involved in diverse nonenzymatic activities such as DNA repair, RNA processing, intracellular signaling, and immune responses [8]. In most cases, MSC functions as a single factor. Once under stress such as viral infections, the MSC components will dissociate from the complex and play their independent roles, although how the MSC components communicate with each other and with free forms of cy-aaRSs requires more investigations.

### 3.1. Inducible Released EPRS Regulates Innate Immune Responses

EPRS is the only bifunctional enzyme for protein synthesis, consisting of GluRS and ProRS via a linker containing 3 WHEP domains. Being an important component of the MSC, EPRS can be sequentially phosphorylated at the residues serine 886 (S886) and S999 in the linker connecting GluRS and ProRS upon the stimulation of IFN-γ, followed by its dissociation from the MSC; the released dual-phosphorylated EPRS, together with NS1-associated protein 1 (NSAP1), phosphorylated ribosomal protein L13a and glyceraldehyde-3-phosphate dehydrogenase (GAPDH), forms an IFN-γ-activated inhibitor of the translation (GAIT) complex [14]. The GAIT complex further represses the translation of multiple IFN-γ-induced inflammatory proteins via its interaction with the eukaryotic initiation factor (eIF) 4G [15,16], providing a novel virus-induced innate immune responsive operon that can be regulated and controlled (Figure 1B).

Transmissible gastroenteritis coronavirus (TGEV) is a CoV that causes a life-threatening disease in pigs. In an early study, one group identified that EPRS and arginyl-tRNA synthetase (ArgRS) preferentially bind to the 3′-end of the TGEV genome, and gene silencing of EPRS leads to reduced TGEV RNA synthesis [17]. Interestingly, recent analysis identified a novel GAIT-like RNA motif at the 3′-end of the TGEV genome, which interacts directly with EPRS and ArgRS. This interaction in turn counteracts the host innate immune response in vitro, and mutations of the viral GAIT-like RNA motif elicit a faster and stronger antiviral response, causing a significant increase in both proinflammatory cytokines and IFN-β [18]. The identification of the TGEV GAIT-like motif and its role in the modulation of the antiviral response provides pivotal implications for novel therapeutic development. More importantly, this feature may be shared by other human CoVs, a study that awaits further studies.

In addition to the EPRS-GAIT system, influenza A virus, a single-stranded negative-sense RNA virus, induces EPRS phosphorylation at a new site of S990, causing its release from the MSC; the released EPRS, instead of forming a GAIT complex, protects MAVS from ubiquitination by poly(rC)-binding protein 2 (PCBP2). The stabilized MAVS further suppresses viral replication and ultimately facilitates antiviral innate immune responses [19] (Figure 1B). Inspiringly, an EPRS-derived peptide shows a potent antiviral activity in vitro against RNA virus vesicular stomatitis virus (VSV) but not DNA virus herpes simplex virus (HSV), suggesting that the antiviral function of EPRS via stabilizing MAVS is specific to RNA viruses [19]. 

### 3.2. ThrRS Stimulates the Activation and Maturation of DCs

Dendritic cells (DC) are a type of antigen-presenting cells (APCs) that link innate and adaptive immunity. Interestingly, a recent study revealed that ThrRS induces the activation and maturation of bone marrow-derived DCs (BMDCs), as well as the primary splenic DCs, which further promote T helper 1 cell (Th1) responses in vitro and in vivo; more importantly, ThrRS-treated DCs exhibit an increased antiviral activity against H1N1 influenza A virus in mice, revealing a novel candidate for a protective adjuvant against respiratory viruses [20]. 

### 3.3. AIMP1 Is Secreted to Stimulate Innate Antiviral Immune Responses

In addition to many intracellular roles, aaRSs can also be secreted extracellularly to exert cytokine-like activities and play important direct or indirect roles in the communication on the cell surface between aaRSs and RNA viruses. 

AIMP1 (also known as p43) is a noncatalytic subunit of the MSC and participates in a variety of cellular processes under physiological and pathological conditions [21,22,23,24,25,26,27]. Notably, AIMP1 can be secreted as an active cytokine from multiple immune cells including macrophages, monocytes, DCs, lymphocytes, natural killing cells, and others; importantly, AIMP1 exerts cytokine activities in proinflammatory and innate immune responses, both in vitro and in vivo [28,29,30,31,32,33,34]. Upon infection of the H1N1 influenza A virus, AIMP1 is upregulated in bronchial epithelial cells, exhibiting a possible role for AIMP1 in response to viral infection [19]. Further microarray analyses of murine BMDCs indicate much lower levels of antiviral genes and innate immune sensors in AIMP1^−/−^ BMDCs compared to the wild-type (WT) BMDCs, suggesting that AIMP1 deficiency impairs innate and adaptive antiviral immunity, in accordance with a higher mortality observed in AIMP1^−/−^ mice when challenged with the H3N2 influenza A virus [35]. Although the precise function of AIMP1 in DCs remains to be proven in the context of the influenza viral infection, its role in regulating antiviral gene expression profiles could at least partially explain the in vivo results.

**Figure 2:**
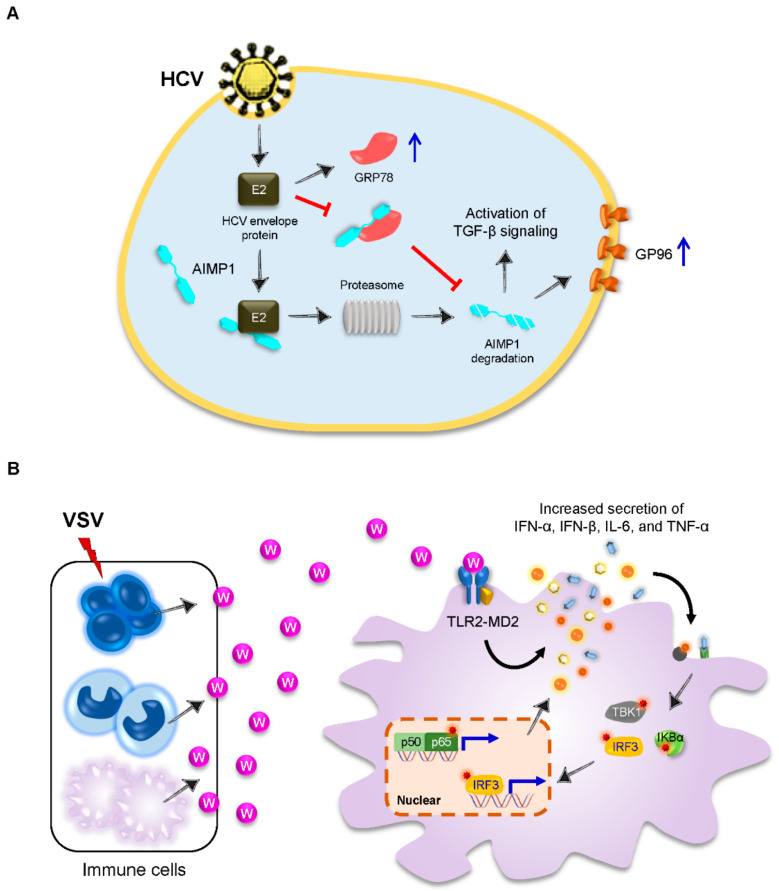
AIMP1 and TrpRS in viral infection. (**A**) Upon HCV infection, HCV envelope protein E2 directly interacts with AIMP1 and induces a ubiquitin-dependent proteasomal degradation of AIMP1. On the other hand, HCV E2 leads to the upregulation of GRP78 (blue arrow) but inhibits the interaction between AIMP1 and its stabilizer GRP78, resulting in AIMP1 degradation. The degradation of AIMP1 leads to activation of TGF-β signaling and increased GP96 expression (blue arrow) on cell surface. AIMP1, aaRS-interacting multi-functional protein 1; GP96, heat shock protein GP96; GRP78, glucose regulated protein 78; HCV, hepatitis C virus; and TGF, transforming growth factor. (**B**) VSV infection induces secretion of TrpRS (purple ball) from immune cells including lymphocytes (left upper cells in dark blue), monocytes (left middle cells in light blue), and macrophages (left lower cells in light purple). The secreted TrpRS elicits innate immune responses and induces antiviral cytokines including IFN-α, IFN-β, IL-6, and TNF-α (particles in different colors). This response is mediated by the TLR4-MD2 receptor on the cell surface. The increased cytokines further induce signaling cascades that result in the phosphorylation (red star) of TBK1, IRF3, and IKBα, which lead to activation of NF-κB (p50/p65) and IRF3 to upregulate the transcription of cytokines. IFN, interferon; IKBα, nuclear factor kappa B inhibitor alpha; IL-6, interlukin-6; IRF3, interferon regulatory factor 3; NF-κB, nuclear factor kappa B; TBK1, TANK binding kinase 1; TLR4-MD2, toll-like receptor 4-myeloid differentiation factor 2; TNF, tumor necrosis factor; TrpRS (W), tryptophanyl-tRNA synthetase; and VSV, vesicular stomatitis virus.

Apart from the influenza virus, the hepatitis C virus (HCV), a single-stranded positive-sense RNA virus, leads to the degradation of AIMP1 in two independent ways: a direct HCV envelope E2-induced ubiquitin-mediated proteasome pathway and an indirect pathway by inhibiting the interaction between AIMP1 and its stabilizer glucose-regulated protein 78 (GRP78), an endoplasmic reticulum chaperone [36]. The degradation of AIMP1 further results in increased transforming growth factor (TGF)-β signaling and cell-surface expression of heat shock protein GP96 (GP96) (Figure 2A), providing a novel cytomembrane communication between AIMP1 and HCV. 

### 3.4. Tryptophanyl-tRNA Synthetase (TrpRS) Elicits Innate Immune Response as a Cytokine

In sepsis patients with a bacterial infection, Kim S and colleagues found increased secretion of TrpRS into the blood [37]. Further mechanical studies revealed that full-length TrpRS (FL-TrpRS) can be secreted rapidly from monocytes into extracellular space and directly binds to macrophages via a toll-like receptor 4 (TLR4)-myeloid differentiation factor 2 (MD2) complex to enhance immune responses against bacterial infection [37]. This groundwork firstly connects TrpRS to the host defense mechanism against infection, even though at that moment it was not clear whether FL-TrpRS should be considered as a new component of innate immunity at an early stage of infection. 

Later on, the Kim S group further demonstrated that TrpRS, like AIMP1, can be rapidly secreted by the immune cells in the early phase of VSV infection and elicits innate immune responses by inducing proinflammatory cytokines and type I IFNs, ultimately inhibiting viral replication in vitro and in vivo [38] (Figure 2B). Thus, this work reveals a novel role of TrpRS as an alarmin to stimulate innate immune responses against viral infection. More importantly, the endogenous factors, like TrpRS, can be developed as adjuvants for vaccination. 

## 4. Host aaRSs Are Employed by RNA Viruses for Invasion

Compared to DNA viruses, RNA viruses have very small genomes that limit their own protein synthesis. Therefore, the host translation machinery is quite essential for the replication and survival of RNA viruses. Being an indispensable component of the host protein translation apparatus, aaRSs are, not surprisingly, the first ones to be employed by RNA viruses.

### 4.1. Lysyl-tRNA Synthetase (LysRS) Is an Essential Host Factor for the Packaging of HIV-1 Virions

**Figure 3:**
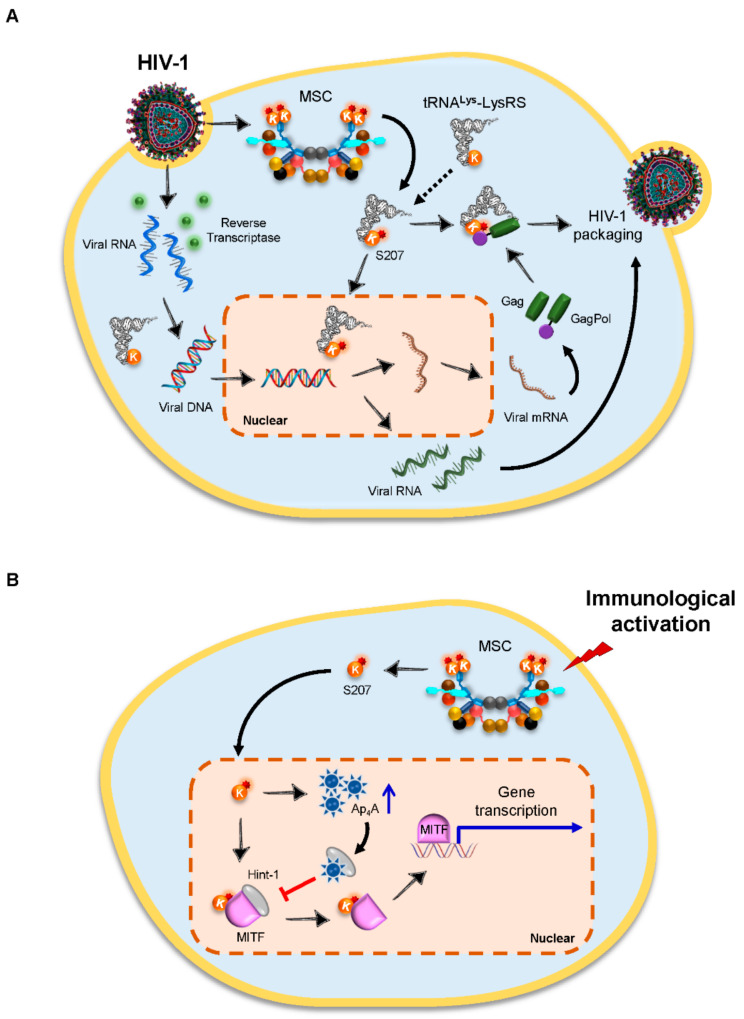
LysRS in HIV-1 infection. (**A**) After HIV-1 infection, the RNA genome of HIV-1 is converted to DNA, which is primed by the host cellular tRNA^Lys^. Human LysRS is also packaged into HIV-1 virions via the interaction with the viral precursor proteins Gag and GagPol. On the other hand, HIV-1 infection triggers the release of LysRS from the MSC via the phosphorylation of serine 207 (S207) (red star) in LysRS. The released LysRS partially translocates to the nucleus. HIV-1, human immunodeficiency virus type 1; LysRS (K), lysyl-tRNA synthetase; and MSC, multi-synthetase complex. (**B**) In immunoglobulin E (IgE)-stimulated mast cells, LysRS is phosphorylated at S207, followed by its release from the MSC. The dissociated LysRS further traffics to the nucleus, where it forms a complex with MITF and Hint-1, as well as produces high levels of Ap**_4_**A (blue arrow). The interaction between Ap**_4_**A and Hint-1 results in the dissociation of Hint-1 from the complex and the activation of MITF-targeted gene transcription. Ap**_4_**A, diadenosine tetraphosphate and MITF, microphthalmia transcription factor.

Human immunodeficiency virus type 1 (HIV-1) is a retrovirus that depends entirely on the host translation machinery. The RNA genome of HIV-1 is converted to DNA before it is integrated into the host genome, and this process is primed by the host cellular tRNAs. During HIV-1 assembly, host-encoded tRNA^Lys^ isoacceptors, tRNA^Lys1,2^ and tRNA^Lys3^, are selectively incorporated into HIV-1 virions, where tRNA^Lys3^ serves as the primer for reverse transcription with its anticodon as a major determinant for the packaging [39]. In addition, human LysRS, the only known protein that specifically recognizes tRNA^Lys^, is incorporated into HIV-1 as well, independently of tRNA^Lys^, via interaction with the viral precursor proteins Gag and GagPol [40] (Figure 3A). Notably, LysRS, but not the viral precursor proteins, is the limiting factor during packaging [41]. The specific inhibition of LysRS results in a decrease in tRNA^Lys^ incorporation, accompanied by reduced tRNA^Lys3^ annealing to HIV-1 genomic RNA and decreased viral infectivity [42]. By contrast, the overexpression of WT LysRS leads to an increase in the viral packaging of both LysRS and tRNA^Lys^ [43]. Interestingly, the increased incorporation of tRNA^Lys^ into HIV-1 virions requires the ability of LysRS in binding to tRNA^Lys^ rather than its ability in aminoacylation, indicating a nonenzymatic role of LysRS upon HIV-1 infection [43]. Further in vitro studies identified that the motif 1 dimerization helix of human LysRS is an essential domain for the packaging of the synthetase into virions [44]. Recently, one study discovered that HIV-1 infection triggers the release of LysRS from the MSC via a specific phosphorylation of S207 in LysRS; the released LysRS partially translocates into the nucleus, and blocking this pathway by the addition of a mitogen-activated protein kinase kinase (MEK) inhibitor in HIV-1-producing cells results in less infectious progeny virions [45]. Interestingly, a phosphomimetic mutant of LysRS (S207D) rescues HIV-1 infectivity and can be packaged into HIV-1 particles, whereas a phosphoablative mutant (S207A) does not [45], suggesting that HIV-1 takes full advantage of the dynamic nature of the host cellular factors to facilitate viral replication (Figure 3A).

It is noteworthy that both cy- and mt-forms of LysRS in humans are encoded by a single gene via an alternative splicing of exon 2 [46]. The two LysRS species share 576 identical AA residues but with the N-terminal sequence of 21 or 49 AA residues distinct [47]. Therefore, the mt-LysRS cannot be excluded by utilizing polyclonal antibodies, as performed in previous studies [40], to identify the presence of LysRS in HIV-1 extracts. Indeed, antibodies specifically targeting mt-LysRS revealed that mt-LysRS is also a cellular source of LysRS detected in the HIV-1 virions [47]. Further analysis determined that the Pol region of the GagPol polyprotein, but not the Gag region, interacts with the catalytic domain of mt-LysRS [48], and no other viral proteins are required in the assembly of the GagPol/mt-LysRS/tRNA^Lys3^ packaging complex [49]. More specifically, mt-LysRS interacts with the transframe and integrase domains of the Pol region in GagPol [50], and their interaction is much more robust and stable than the previously reported interaction between cy-LysRS and Gag [51].

It is important to note that the HIV-1 genome contains a tRNA anticodon-like element (TLE) that mimics the anticodon loop of tRNA^Lys^. This molecular mimicry allows a specific direct binding of TLE to the human LysRS, even in the presence of tRNA^Lys3^ [52]. The interaction between TLE and human LysRS also facilitates the release of tRNA^Lys3^ from the synthetase prior to reverse transcription [53], thus providing an alternative mechanism of HIV-1 to increase the efficiency of tRNA primer annealing. Remarkably, this tRNA mimicry is conserved across distinct HIV-1 subtypes [54]. 

In addition to its roles in HIV-1 packaging, LysRS can also activate several genes by synthesizing diadenosine tetraphosphate (Ap**_4_**A) hydrolase. In immunoglobulin E (IgE)-stimulated mast cells, LysRS is phosphorylated at S207, followed by its dissociation from the MSC; the released LysRS further traffics to the nucleus, where it forms a complex with microphthalmia transcription factor (MITF) and Hint-1; in contrast, LysRS produces Ap**_4_**A, which further interacts with Hint, leading to the dissociation of Hint-1 from the complex and the subsequent activation of MITF-targeted gene transcription [55,56] (Figure 3B). These findings, together with the interaction between LysRS and Gag/GagPol, highlight a crucial role of LysRS in the host immune responses under infectious stimulus.

In addition to LysRS, aspartyl-tRNA synthetase (AspRS) also participates in HIV replication, although the detailed mechanisms remain unknown [57]. Given their key roles in protein translation and synthesis, more aaRSs will be discovered in the replication and packaging of viruses.

### 4.2. Glycyl-tRNA Synthetase (GlyRS) Regulates Viral Translation Initiation via the IRES Element

In eukaryotic cells, the translation initiation of most messenger RNAs (mRNAs) occurs in a cap-dependent manner that requires ribosomal scanning of their 5′ untranslated region (5′-UTR). However, in several groups of RNA viruses, such as picornaviruses, mRNAs initiate translation in a cap-independent mechanism by utilizing an internal ribosome entry site (IRES). IRES elements are specific RNA structures located at the 5′-UTR of the mRNA that recruit components of the host translation machinery to regulate translation initiation [58]. The presence of an IRES in the viral RNA facilitates efficient viral translation initiation and inhibits host cell protein synthesis, an important mechanism of counteracting host defense. 

**Figure 4:**
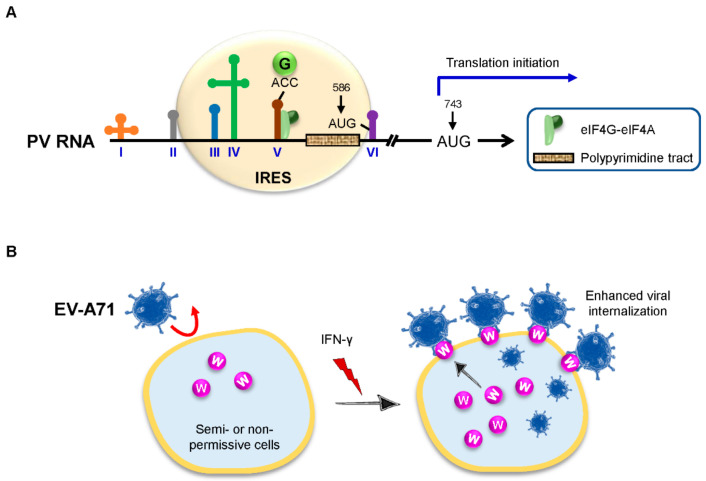
GlyRS and TrpRS in viral infection. (**A**) Stem-loop domains of PV RNA are numbered by Roman numbers. The PV IRES covers the region from domain II through domain VI. The non-initiator of AUG at 586, the authentic AUG at 743 as well as the polypyrimidine tract are shown. GlyRS (green ball) binds to the domain V of PV IRES, adjacent to the binding site of the eIF4G-eIF4A complex. The upper part of domain V mimics the anticodon stem-loop of tRNA^Gly^ with the anticodon ACC. The binding site of the eIF4G-eIF4A complex on domain V is based on a previous study [61]. eIF, eukaryotic initiation factor; GlyRS (G), glycyl-tRNA synthetase; IRES, internal ribosome entry site; and PV, poliovirus. (**B**) EV-A71 semi- or non-permissive cells have low or no surface expression of TrpRS (purple ball), which decreases EV-A71 infection (red curved arrow, left). By contrast, IFN-γ induces the expression and membrane translocation of TrpRS, which in turn sensitizes cells to viral infection via the direct interaction with EV-A71, ultimately leading to enhanced viral internalization (right). EV-A71, enterovirus A71; IFN, interferon; and TrpRS (W), tryptophanyl-tRNA synthetase.

Poliovirus (PV) is a prototype member of the picornaviruses that is mainly involved in motor neurons, leading to paralytic poliomyelitis caused by the degeneration and lysis of neuron cells. Although PV vaccines have been widely deployed to control the disease, the events in the course of internal entry of PV onto the IRES elements are not yet clear. Based on a systematic screening for factors involved in this process, GlyRS was identified as a new participant required for PV RNA translation. GlyRS specifically binds to the domain V of the PV IRES, which mimics the anticodon stem-loop of tRNA^Gly^, resulting in the high affinity and specificity of this interaction [59] (Figure 4A).

Of interest, dominant mutations in GlyRS can cause Charcot-Marie-Tooth disease (CMT), the most common inherited neuromuscular disorder presented as loss of muscle tissue and touch sensation in body extremities. Defective neurite localization and distribution in cells were found upon CMT-causing GlyRS mutation, implying impaired mRNA function [60]. This finding highlights a noncanonical role of GlyRS in neural diseases, along with its role in PV RNA translation initiation, which needs to be taken into account in viral infections of the central nervous system. 

### 4.3. TrpRS Functions as a Cellular Entry Factor for Enteroviruses 

In addition to the cytokine-like role discussed above, TrpRS also functions as a cellular entry factor for enteroviruses (EV). EV-A71 is a major causative agent of hand, foot, and mouth disease (HFMD), which has been periodically reported worldwide, especially in East Asia. Vaccines for EV-A71 infection are now available in China, but no effective anti-EV-A71 therapy is available at present. Other than EV-A71, there are many common human EVs such as HFMD-causing coxsackievirus A16 (CV-A16) and EV-D68, which causes severe respiratory illness. 

In a recent study, TrpRS was revealed as an inducible entry factor for EV-A71. IFN-γ stimulation leads to an increase in the surface expression of TrpRS, and the direct interaction between TrpRS and EV-A71 is required for the host cell entry [62] (Figure 4B). In mouse models, the overexpression of human TrpRS sensitizes murine cells to EV-A71 infection [62]. In addition to EV-A71, infections with CV-A16, EV-D68, and other human EVs also require the expression of TrpRS, suggesting that TrpRS has a broad role in virus entry [62]. These findings raise the possibility of developing specific antibodies or recombinant proteins by blocking the accessibility of TrpRS for treating EV-associated diseases or complications. On the other hand, these findings will stimulate future efforts to delineate the roles of aaRSs in cell entry for other RNA viruses. 

## 5. Concluding Remarks and Future Directions

Although the roles of aaRSs in RNA viral infection are incompletely understood, the functional plasticity is remarkable for aaRSs. During evolution, the selective pressure makes them gain diverse biological functions, equally essential but outside of their historical roles. These nonenzymatic functions of aaRSs seem to be even greater in humans, especially when challenged by viruses. As discussed herein, aaRSs exert multiple biological functions upon an RNA viral hijack, and some of them have nothing to do with protein synthesis. These functions include (i) the formation of new complexes; (ii) interaction with intracellular factors; (iii) intranuclear function; (iv) virus entry, and (v) extracellular secretion (Table 1).

In fact, the noncanonical roles of aaRSs in infectious diseases are being unveiled at a rapid pace. The exciting work in this field may lead to new therapies for human infections. For example, the natural product inhibitors of aaRSs have been successfully identified as a new class of antibiotics or antiparasitics decades ago [63,64]. Nonetheless, progress in aaRS-based therapeutics against viruses seems to be lagging far behind. One notable example is halofuginone, a potent orally-available EPRS antagonist, which suppresses both alphaviruses and flaviviruses by preventing aminoacylation [65]. Although inhibiting host translation may lead to toxic effects, proper dosing or synergy with other antiviral agents may provide a feasible way to combat present and future epidemic viruses where therapeutics would not be readily available.

One of the major challenges in aaRS-based drug discovery is the need to capture a detailed picture of aaRSs upon infection. To achieve this, efforts in the accumulation of robust high-throughput screening as well as aaRS structural and mechanistic information are urgently needed. For example, an overall downregulation of cy-aaRSs was identified in clinical samples from COVID-19 patients and SARS-CoV-2-infected cells [5], but the molecular mechanisms underlying the viral infection and propagation are incompletely elucidated. Although similar expression trends between cy-aaRSs and cytoplasmic translation, and ribosome proteins were observed, other essential information such as the cellular tRNA populations and the charging levels of tRNAs are still missing [5]. In this sense, it is very likely that the overall decrease in cellular mRNAs and translational efficiency is attributed to the downregulation of cy-aaRSs upon SARS-CoV-2 infection, although we cannot exclude the possibility that cy-aaRSs play noncanonical roles during this process.

Likewise, an upregulation of 14 aaRSs was found following an infection of flavivirus in the brain [66], and the same increase of aaRSs was also observed in response to West Nile virus infection in Vero cells [67]. However, it is still unclear what the roles of aaRSs play during virus entry and replication. In this regard, we can probably seek inspiration from an excellent study on a single-stranded positive-sense RNA virus, a tRNA-like structure (TLS) found at the 3′-end of the Turnip Yellow Mosaic Virus (TYMV) [68]. Based on the crystal structure of the TYMV TLS RNA, researchers found that the RNA is shaped like a tRNA and also allows other activities by switching its conformation. As a result, the TLS structure is bifurcated, with one mimicking tRNA to drive aminoacylation and the other enabling additional functions and interactions. This work inspires synthetic efforts to fully understand the structural differences of pathogens and host enzymes, such as aaRSs, during viral infection, thus providing new opportunities for therapeutic efforts to target a certain function involved in pathology while sparing physiological housekeeping roles.

Last but not least, aaRSs could be developed as therapeutic cytokines or adjuvants for vaccination, since some of the aaRSs, such as AIMP1 and TrpRS, do have cytokine-like activities. Thus, the secreted aaRSs have great potential to boost defensive immunity against primary viral infections. On the other hand, blocking the secretion of aaRSs or neutralizing the secreted aaRSs could also alleviate virus-induced inflammation. This possibility, together with the aforementioned opportunities for therapies, leads to the necessity of more detailed characterization of aaRSs with huge efforts exerted in future studies.

## Figures and Tables

**Table 1:** Roles of aaRSs and AIMPs in RNA viral infection.

aaRS		RNA Virus	Effect	Interactor	Mechanisms	Ref.
Regulate innate immune responses	mt-ThrRS	SARS-CoV-2	Antivirus	SARS-CoV-2 M protein	-	[5]
	EPRS, ArgRS	TGEV	Inhibit innate immune responses	GAIT-like RNA motif of TGEV genome	Binding to GAIT-like RNA motif at 3′-end of the TGEV genome to counteract the host innate immune response	[18]
	EPRS	H1N1 influenza A; VSV	Antivirus	PCBP2	Stabilizing MAVS from PCBP2-mediated ubiquitination to facilitate antiviral function	[19]
	ThrRS	H1N1 influenza A	Antivirus	-	Inducing DC maturation and IL-12 production via promoting Th1 responses	[20]
	AIMP1	H3N2 influenza A	Antivirus	-	Activating MAPK signaling and Th1 polarization in LPS-treated BMDCs	[35]
	AIMP1	HCV	-	HCV envelope protein E2	Inducing AIMP1 degradation to enhance TGF-β signaling and cell surface expression of GP96	[36]
	TrpRS	VSV	Antivirus	TLR4-MD2	Inducing proinflammatory cytokines and type I IFNs	[37,38]
Facilitate viral infection	LysRS	HIV-1	Virus packaging	Gag, GagPol	Activating MEK pathway; translocating to the nucleus	[45]
	LysRS	HIV-1	Annealing of tRNA to viral RNA	TLE of HIV-1 genome	Binding to TLE near the primer-binding site within the HIV-1 genome to increase the efficiency of tRNA^Lys3^ primer annealing	[52,53]
	GlyRS	PV	RNA translation initiation	PV IRES	Binding to the domain V of the PV IRES which mimics the anticodon stem-loop of tRNA^Gly^	[59]
	TrpRS	EV-A71	Virus entry	EV-A71	Binding to EV-A71 viral proteins to enhance viral internalization	[62]

AIMP1, aaRS-interacting multi-functional protein 1; ArgRS, arginyl-tRNA synthetase; BMDCs, bone marrow-derived dendritic cells; DC, dendritic cell; EPRS, glutamyl-prolyl-tRNA synthetase; EV-A71, enterovirus A71; GAIT, IFN-γ-activated inhibitor of translation; GlyRS, glycyl-tRNA synthetase; GP96, heat shock protein GP96; HCV, hepatitis C virus; HIV-1, human immunodeficiency virus type 1; IFN, interferon; IL-12, interleukin-12; IRES, internal ribosome entry site; LPS, lipopolysaccharide; LysRS, lysyl-tRNA synthetase; M protein, main protease protein; MAPK, mitogen-activated protein kinase; MAVS, mitochondrial antiviral signaling protein; MEK, mitogen-activated protein kinase kinase; mt-ThrRS, mitochondrial threonyl-tRNA synthetase; PCBP2, poly(rC)-binding protein 2; PV, poliovirus; Ref., reference(s); TGEV, transmissible gastroenteritis coronavirus; TGF-β, transforming growth factor β; Th1, T helper 1 cell; TLE, tRNA anticodon-like element; TLR4-MD2, toll-like receptor 4-myeloid differentiation factor 2; TrpRS, tryptophanyl-tRNA synthetase; and VSV, vesicular stomatitis virus.

## Data Availability

Not applicable.
